# Awareness of stroke among patients with chronic kidney disease on hemodialysis: a cross-sectional study

**DOI:** 10.1590/1516-3180.2022.0644.R1.24042023

**Published:** 2023-07-31

**Authors:** Orlando Vieira Gomes, Manoel Pereira Guimarães, Bárbara Maria Batista Barbosa, Christielle Lidianne Alencar Marinho, Jandir Mendonça Nicacio, Matheus Pereira Barreira, Mateus de Sousa Rodrigues, Leonardo Fernandes e Santana, Ubiracé Fernando Elihimas, Paulo Adriano Schwingel

**Affiliations:** I MD, MSc. Nephrologist and Assistant Professor, University Hospital, School of Medicine, Universidade Federal do Vale do São Francisco (UNIVASF), Petrolina (PE), Brazil.; II Undergraduate Medicine Student, School of Medicine, Universidade Federal do Vale do São Francisco (UNIVASF), Petrolina (PE), Brazil.; III MD. Physician and Specialist in Internal Medicine, University Hospital, School of Medicine, Universidade Federal do Vale do São Francisco (UNIVASF), Petrolina (PE), Brazil.; IV MSc. Nurse and Doctoral Student, Health Sciences Program, Universidade de Pernambuco (UPE), Recife (PE), Brazil.; V MD, MSc. Hematologist and Assistant Professor, University Hospital, School of Medicine, Universidade Federal do Vale do São Francisco (UNIVASF), Petrolina, Brazil.; VI Undergraduate Medicine Student, School of Medicine, Universidade Federal of Vale do São Francisco (UNIVASF), Petrolina (PE), Brazil.; VII MD. Physician and Neurosurgery Resident, University Hospital, School of Medicine, Universidade Federal do Vale do São Francisco (UNIVASF), Petrolina (PE), Brazil.; VIII MD. Physician and Internal Medicine Resident, University Hospital, School of Medicine, Universidade Federal do Vale do São Francisco (UNIVASF), Petrolina (PE), Brazil.; IX MD, MSc, PhD. Nephrologist, Nephrology Service, University Hospital, Universidade Federal de Pernambuco (UFPE), Recife (PE), Brazil.; X PhD. Sports Physiologist and Associate Professor, Human Performance Research Laboratory, Universidade de Pernambuco (UPE), Petrolina (PE), Brazil.

**Keywords:** Renal insufficiency, chronic, Stroke, Awareness, Renal dialysis, Renal replacement therapy, Chronic kidney insufficiency, Chronic kidney disease, Knowledge about stroke, Hemodialysis, Kidney replacement therapy

## Abstract

**BACKGROUND:**

Stroke is a major cause of mortality worldwide. Renal dysfunction is an important risk factor for stroke. Brazilian studies on stroke knowledge are generally population based. Studies stratifying stroke knowledge according to comorbidities are rare. Scientific data are essential to guide the awareness of stroke.

**OBJECTIVE:**

To assess stroke knowledge in patients with chronic kidney disease (CKD) on hemodialysis.

**DESIGN AND SETTING:**

Cross-sectional analytical study of patients with CKD on hemodialysis in north-eastern Brazil.

**METHODS:**

A self-administered questionnaire survey on stroke awareness was administered to patients with CKD on hemodialysis between April and November 2022. The chi-square test and other descriptive statistics were used. Univariate and multivariate analyses were performed using logistic regression.

**RESULTS:**

A total of 197 patients were included in the analysis. The Brazilian acronym for stroke was used by 53.5% of the participants. Less than 10.0% of the sample showed optimal decision-making ability regarding stroke. Of the participants, 29.9% knew at least one risk factor and one symptom; however, this was considered as having below the minimum capacity because they did not know the emergency service call number. In the analysis adjusted for income and education, females (odds ratio [OR], 0.40%; 95% confidence interval [CI], 0.20-0.82), older patients (OR, 0.24%; 95% CI, 0.09-0.63) and having at most one comorbidity (OR, 0.48%; 95% CI, 0.23-0.98) were factors for lower levels of knowledge or ideal decision-making capacity against stroke.

**CONCLUSIONS:**

Patients on hemodialysis, especially women and older people, have little knowledge about stroke.

## INTRODUCTION

Stroke is the second leading cause of death worldwide, accounting for approximately 11% of total mortality in 2019.^1^ Among Latin American countries, Brazil has one of the highest mortality rates for stroke. Despite the downward trend in the mortality rates in recent years, this decline is not evenly distributed across all regions of the country, as the northeastern region of Brazil continues to have high rates.^2^

Patients with chronic kidney disease (CKD) are at increased risk for stroke.^3^ CKD is associated with more severe stroke, provides higher mortality in this group of individuals, increases the incidence of silent stroke and cognitive impairment.^4^ The risk factors for developing CKD itself, such as hypertension and diabetes, increase the likelihood of stroke. In addition, patients with CKD have a higher risk of developing carotid artery disease and heart failure.^3,4^ Furthermore, people with CKD have an increased risk of atrial fibrillation,^5^ which in turn increases the risk of stroke.^6^

Studies show that patients with CKD have a 9.1% higher risk of having a stroke,^7^ and this risk increases with the progression of renal dysfunction.^3^ However, Brazilian studies on stroke knowledge typically focus on the general population and lack specific insights on comorbidity-related knowledge.^8,9^ Although CKD is a prevalent pathology and patients with this comorbidity are at greater risk of a vascular event,^7^ there is a significant gap in knowledge regarding stroke recognition for this patient population. Therefore, it is critical to conduct scientific research specifically on patients with nephropathy to improve their awareness of stroke and establish appropriate measures for prevention and treatment.

In Brazil, knowledge about stroke in the general population is low,^8–10^ and it is believed that this is also true in the CKD population. It is also noteworthy that failure to recognize these signs and symptoms delays seeking medical care, which negatively affects poststroke treatment and reduces the likelihood of recovery.^11^

## OBJECTIVE

This study aimed to determine the level of knowledge about stroke in a population with CKD at a dialysis center in north-eastern Brazil. Consequently, we investigated the ability of the study population to recognize a stroke and the correct triggering of emergency services.

## METHODS

### Study design and population

This was a cross-sectional analytical study conducted in a dialysis center located in the city of Juazeiro, state of Bahia, Brazil, following the recommendations of the statement Strengthening the Reporting of Observational Studies in Epidemiology.^12^ In this study, data were collected between April and November 2022.

The study included patients with CKD on hemodialysis who voluntarily agreed to participate in the research and answer the data collection instrument using a semi-structured questionnaire (**Attachment 1**). The following inclusion criteria were established: (1) age of > 18 years, (2) have been on treatment for > 3 months, and (3) no known history of cognitive impairment. Those who did not complete the questionnaire were excluded.

### Variables

The questionnaire used in the study had already been used in another study with a different population.^10^ It was based on a literature search that included other studies that also examined the level of knowledge about stroke in their respective target populations.^8,9^ Each participant was asked to answer sociodemographic questions that included information about sex, age, family income, and education, as well as questions about stroke knowledge. In addition, the history of comorbidities reported by each participant was examined.

The stroke questionnaire consisted of four questions: (1) Do you know what a stroke is? (2) Can you name at least three signs or symptoms of stroke? (3) Can you identify at least three risk factors for stroke? (4) What is the phone number of the emergency medical service in Brazil?

### Level of knowledge about stroke

In this study, stroke knowledge was assessed based on the ability to make decisions when faced with a stroke. This decision-making was categorized into three levels: (1) ideal (able to recognize three symptoms and three risk factors and know how to call the emergency medical service), (2) minimum required (able to recognize one symptom and one risk factor and know how to call the emergency medical service), and (3) below minimum (none of the aforementioned characteristics met).

### Sample size and statistical analysis

A total of 443 patients were registered at the dialysis center. A minimum sample size of 192 participants was sufficient to achieve a precision of 10% around our estimate of stroke knowledge of 43.9%^8^ with a confidence level of 99.9% assuming a non-response rate of 15%.

The data obtained were double entered into the computer program SPSS (IBM, version 16.0.2, 2008, United States) to check for consistency and range. Descriptive statistical analysis was performed, in which categorical variables were presented as absolute and relative frequencies, whereas continuous variables were presented as means and standard deviations (SDs) after determining the normality of the data using the Kolmogorov-Smirnov test. The association between baseline characteristics and stroke knowledge was determined by univariate analysis using Pearsons chi-square test (χ^2^). Variables with P ≤ 0.20 in these analyses were selected for multivariate analysis by logistic regression performed with the stepwise technique to identify the predictors of decision-making capacity against stroke. The unadjusted and adjusted odds ratios (OR) and confidence intervals of 95% (95% CI) were calculated. Statistical analyses were two-tailed, and statistical significance was defined as P < 0.05.

### Ethical considerations

This study was approved by the Ethics Committee for Research Involving Human Subjects of the Faculdade de Integração do Sertão (protocol no. 5.361.385), issued on April 20, 2022. Throughout this study, the ethical principles of the Declaration of Helsinki (1964) were adhered to by Resolutions 466/2012 and 510/2016 of the Brazilian National Health Council. All volunteers who agreed to participate in the study signed an informed consent form before the interview.

## RESULTS

The study sample comprised of 197 participants. Of these, 106 (53.8%) were men. The mean age (± SD) was 50.77 (± 16.97) years. A total of 105 participants (53.5%) recognized the Brazilian acronym for cerebrovascular accidents. The sociodemographic profile of the sample and prevalence of each response to stroke knowledge are presented in Table 1.

**Table 1:** Demographics and stroke knowledge mentioned by participants (n = 197)

Variables		n	%
**Sex**			
	Male	106	53.8
	Female	91	46.2
**Age**			
	18-39 years	38	19.3
	40-59 years	78	39.6
	≥ 60 years	81	41.1
**Per capita income**			
	Less than 1 minimum wage (< US$ 200.00)	121	61.4
	From 1 to 2 minimum wages (U$ 200.00-400.00)	53	26.9
	≥ 3 minimum wages (> U$ 400.00)	23	11.7
**Educational attainment**			
	Illiterate or elementary school not completed	72	36.5
	Elementary school and/or middle school not completed	47	23.9
	Middle school and/or high school not completed	28	14.2
	High school and/or higher education not completed	40	20.3
	Higher education	10	5.1
**I know what a stroke is**			
	Yes	105	53.3
	No	92	46.7
**I know some signs and symptoms of stroke**			
	None	48	24.4
	1	55	27.9
	2	63	32.0
	≥ 3	31	15.7
**I know some risk factors for stroke**			
	None	72	36.5
	1	47	23.9
	2	40	20.3
	≥ 3	38	19.3
**I know the EMS telephone number**			
	Yes	70	35.5
	No	170	64.5

EMS = Emergency Medical Services.

**Figure 1:**
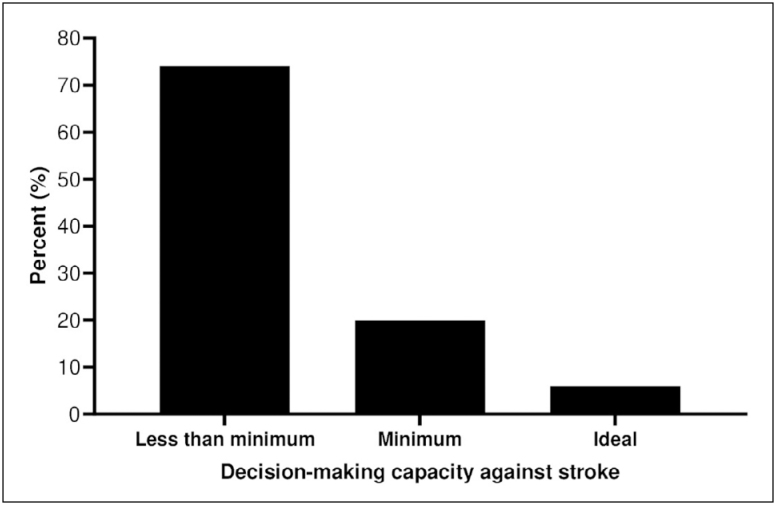
Decision-making capacity against stroke.

The decision-making capacities of patients with stroke are shown in Figure 1. In the population studied, most of the participants presented knowledge below the minimum, according to the established criteria. Less than 10.0% of the sample had an ideal decision-making capacity. It is noteworthy that 29.9% of the participants knew at least one risk factor and one symptom; however, this knowledge was classified as below the minimum because they did not know the emergency service call number.

In the unadjusted investigation, women had an OR of 0.49 (95% CI, 0.25-0.95) for minimal or ideal knowledge of stroke compared with the reference (men) in the logistic regression (Table 2). In addition, the age of > 60 years had an OR of 0.33 (95% CI, 0.140.83) for minimal or ideal knowledge about stroke compared with the age group between 18 and 39 years (Table 2).

**Table 2:** Odds ratios of the association between baseline characteristics and stroke knowledge

Variables		n (%)	Minimum or ideal decision-making capacity against stroke	
			Crude OR (95% CI)	OR adjusted* (95% CI)
**Sex**				
	Male	106 (53.8%)	1	1
	Female	91 (46.2%)	0.49 (0.25-0.95)	0.40 (0.20-0.82)
**Age**				
	18-39 years	38 (19.3%)	1	1
	40-59 years	78 (39.6%)	0.96 (0.42-2.18)	0.70 (0.29-1.69)
	≥ 60 years	81 (41.1%)	0.33 (0.14-0.83)	0.24 (0.09-0.63)
**Per capita income**				
	≥ 3 minimum wages (> U$ 400.00)	23 (11.7%)	1	–
	From 1 to 2 minimum wages (U$ 200.00-400.00)	53 (26.9%)	0.96 (0.34-2.70)	–
	Less than 1 minimum wage (< US$ 200.00)	121 (61.4%)	0.49 (0.19-1.28)	–
**Educational attainment**				
	Higher education	10 (5.1%)	1	–
	High school and/or higher education not completed	40 (20.3%)	0.81 (0.20-3.35)	–
	Middle school and/or high school not completed	28 (14.2%)	0.83 (0.19-3.67)	–
	Elementary school and/or middle school not completed	47 (23.9%)	0.57 (0.14-2.37)	–
	Illiterate or elementary school not completed	72 (36.5%)	0.24 (0.06-1.01)	–
**Comorbidities associated with CKD**				
	≥ 2	85 (43.1%)	1	1
	At least 1	28 (14.2%)	0.65 (0.34-1.24)	0.48 (0.23-0.98)

OR = odds ratio; CI = confidence interval; CKD = chronic kidney disease.

* Adjusted for per capita income and educational attainment.

Moreover, in the analysis adjusted for income and education, it was found that in addition to female sex and the age of > 60 years, having at most one comorbidity was associated with having a less minimal or ideal capacity to cope with a stroke.

## DISCUSSION

Our results show that in the population with CKD on hemodialysis, knowledge about stroke is generally below the minimum. This low level of knowledge about the recognition of stroke, in both quantitative and qualitative terms, is also considered insufficient by the general population in Brazil^8–10^ and some other countries.^13–15^ In a cross-sectional study conducted between 2011 and 2012 in Poland with a sample of 1,134 participants, more than 40% of the study population could not identify any stroke symptoms, and less than 40% were able to identify at least two risk factors for stroke.^15^

Importantly, our results may have been influenced by the socioeconomic profile of the sample. Most participants had a family income of less than $200.00, and only 5.1% had completed a college education. These results could be related to the fact that the study was conducted in Northeast of Brazil, which is characterized by large socioeconomic differences from other regions of the country. Many cities in this region are characterized by a low Human Development Index (HDI) and high indicators of illiteracy, infant mortality, and poverty.^16^

In similar studies conducted in the southeastern region of Brazil, a region with a higher HDI and lower poverty index than the region in which this study was conducted, researchers also found that the samples’ knowledge of stroke was low.^8,9^ Nevertheless, in the sample by Gomes et al., 35.0% knew at least three risk factors for stroke, 17.9% could name at least three signs or symptoms of stroke, and 33.6% knew how to call the emergency services. Notably, 25.2% of the participants had completed a college education.^8^

In a recent study composed of a population of high school students from public schools in the northeastern region of Brazil, the results were virtually the same: only 10.0% ofthe students knew how to ideally act in a stroke situation, and 80.0% did not have the minimum knowledge of how to act in a stroke situation.^10^ This deplorable scenario may reflect the lack of investment by public administrators in awareness and training to recognize medical emergencies.

Some highlights of the results of the adjusted analysis are that women and older people know less about stroke. We believe that the fact that female participants reported lower knowledge is an occasional finding because other studies,^17–21^ including Brazilian samples,^17,20^ have shown that being female increases the likelihood of recognizing the signs of stroke.

As for the older population, a study with a sample of 200 elderly Egyptians already known to have hypertension showed that more than half of them had inadequate knowledge about stroke, although almost a quarter of them had a history of stroke.^22^ In contrast, a study conducted with the European population showed that the older population can recognize the signs and symptoms of stroke.^23^ This suggests that the reason for the lower knowledge of the older adults who participated in our study is the lower educational level of this population.

Notably, participants with two or more comorbidities associated with CKD had a better decision-making capacity against stroke. The main hypothesis is that as the number of comorbidities increases, the frequency of physician care and other health professionals also increases. This would lead to better access to information. A European study with a Norwegian sample showed that cardiac patients had broader knowledge of stroke symptoms and risk factors than the general population.^24^

Our study has limitations because it was performed in only one dialysis center. The lack of studies examining knowledge of stroke in the population with CKD makes comparisons and further analyses of the subject difficult. In addition, most participants in our study had a low income and education, which may not faithfully represent the Brazilian population with end-stage renal disease on dialysis.

It is noteworthy that 81.6% of patients on dialysis in Brazil receive treatment financed by the Brazilian public health system.^25^ However, individuals in this population are typically characterized by lower income and education levels, which may lead to a lack of awareness of stroke. These findings highlight the urgent need for increased investment in health education campaigns aimed at raising awareness and providing training to recognize medical emergencies.

The findings of this study have significant implications. By gaining a better understanding of the relationship between comorbidities and the risk of stroke in patients on dialysis, healthcare providers can personalize their interventions and support for this population. Moreover, health authorities can use these findings to inform policy decisions regarding the allocation of resources and investment in health education campaigns aimed at improving outcomes for patients on dialysis.

Health education campaigns are likely to help change this situation, as research has shown a positive correlation between the presence of comorbidities, increased access to health services, and knowledge about stroke.

## CONCLUSION

Patients on dialysis have an increased risk of stroke compared with the general population; however, knowledge about this condition is frequently insufficient, particularly among female and older people.

## Appendix

**Attachment 1:** English version of the questionnaire

Questions on sociodemographic issues and stroke knowledge:	
**1. Sex:**	
	_ Male
	_ Female
	_ Rather not answer
**2. Age: ___**	
**3. Per capita income: ____**	
**4. Educational attainment**	
	__ Illiterate or elementary school not completed
	__ Elementary school and/or middle school not completed
	__ Middle school and/or high school not completed
	__ High school and/or higher education not completed
	__ Higher education
**5. Do you know what a stroke is?**	
**6. Can you indicate at least three signs or symptoms of stroke?**	
**7. Can you indicate at least three risk factors for stroke?**	
**8. What is the telephone number of the emergency medical services in Brazil?**	
**9. Do you have any comorbidities? How many?**	
